# O1-1 Footfall trends on Irish walking trails before and during the COVID-19 pandemic

**DOI:** 10.1093/eurpub/ckac094.001

**Published:** 2022-08-29

**Authors:** Dylan Power, Barry Lambe, Niamh Murphy

**Affiliations:** Department of Sport and Exercise Science, Waterford Institute of Technology, Waterford, Ireland

**Keywords:** Walking, monitoring, trails, footfall

## Abstract

**Background:**

Organisations operating at multiple levels of the physical activity and outdoor recreation ecosystem in Ireland collect data pertaining to recreational trail use, yet despite its potential utility, there is no centralised surveillance system for Irish walking trails. Due to the little effort to collate data, recreational walking trail usage in Ireland during the COVID-19 pandemic is unknown. The aims of this study are twofold. Firstly, this study aims to compare trends in footfall count data on Irish trails prior to, and during, the period following the outbreak of COVID-19. Secondly, this study aims to triangulate findings from footfall count data with openly available mobility data.

**Methods:**

This descriptive study analysed changes in footfall counts gathered from passive infrared sensors on 33 of Ireland's walking trails between January 2019 and December 2020. The relationship between Google Community Mobility Report (GCMR) data and footfall counts was analysed to corroborate trends in footfall data.

**Results:**

Overall, total footfall increased by 6% (p = 0.024) between 2019 and 2020 on trails included in this analysis. Notably, mean trail usage was between 26% and 47% higher (p = 0.002) in October-December 2020 than during the same period in 2019. On average, trails >2km from an urban area had higher footfall during all periods of movement restrictions.

**Conclusions:**

The conclusions of this study are twofold. Firstly, recreational walking trail use increased during the COVID-19 pandemic, especially on trails closer to urban areas. The increase reported here poses questions relating to the potential positive legacy which the pandemic period may have had on recreational trail use. Secondly, combining multiple data sources can provide trail managers with more detailed representations of trail usage. However, there are currently little efforts to harmonise data. Future research should examine the multi-level determinants of trail use to inform context specific trail promotion campaigns. Furthermore, investigating novel ways to coordinate heterogeneous datasets pertaining to recreational walking may prove fruitful for the fields of outdoor recreation and physical activity research.

